# Diagnostic value of signs, symptoms and diagnostic tests for diagnosing pneumonia in ambulant children in developed countries: a systematic review

**DOI:** 10.1038/s41533-018-0104-8

**Published:** 2018-10-26

**Authors:** Marjolein J. C. Schot, Anne R. J. Dekker, Wesley G. Giorgi, Rogier M. Hopstaken, Niek J. de Wit, Theo J. M. Verheij, Jochen W. L. Cals

**Affiliations:** 1 Julius Center for Health Sciences and Primary Care, University Medical Center Utrecht, Utrecht, The Netherlands; 2 Department of Family Medicine, CAPHRI Care and Public Health Research Institute, Maastricht University, Maastricht, The Netherlands; 3 Star-SHL, Etten-Leur, The Netherlands

## Abstract

Identifying a child with pneumonia in the large group of children with acute respiratory tract infections can be challenging for primary care physicians. Knowledge on the diagnostic value of specific signs and symptoms may guide future decision rules and guidelines for clinicians. We aimed to identify and systematically review available evidence for the diagnostic value of signs, symptoms, and additional tests to diagnose pneumonia in children in an ambulatory setting in developed countries. We conducted a systematic review, searching in the electronic databases of PubMed and Embase. Quality assessment of studies was done using the QUADAS-2 criteria. After data extraction from selected studies, we calculated and summarized test characteristics (sensitivity, specificity, negative and positive predictive values) of all available signs, symptoms, additional laboratory tests, and chest ultrasonography. The original search yielded 4665 records, of which 17 articles were eligible for analysis: 12 studies on signs and symptoms, 4 on additional laboratory tests, and 6 on ultrasonography. All included studies were performed in a secondary care setting. Risk of bias was present in the majority of studies in the domain of patient selection. Prevalence of pneumonia varied from 3.4% to 71.7%. The diagnostic value of the available 27 individual signs and symptoms to identify pneumonia was low. In a low prevalence setting, (4 studies, pneumonia prevalence <10%) clinically ill appearance of the child and oxygen saturation <94% can aid a physician. In a high prevalence setting (10 studies, pneumonia >10%), additional diagnostic tests such as oxygen saturation, C-reactive protein, and white blood cell count are more promising. Chest ultrasonography showed high diagnostic value in settings with higher prevalence of pneumonia. Single signs and symptoms from medical history and physical examination or individual additional diagnostic tests are insufficient to diagnose pneumonia in ambulant children. Very few diagnostic studies are conducted in settings with low prevalence of pneumonia. Future research in low prevalence settings should focus on the diagnostic value of the combination of clinical features and additional testing possibly using meta-analysis of individual data.

## Introduction

Acute respiratory tract infections in children are very common and one of the most important reasons to consult the general practitioner (GP), pediatrician, or emergency physician in developed countries.^1–3^ Most children will suffer from a non-serious, self-limiting infection, but it is important to adequately identify a child with pneumonia. Even in high-income countries including the UK and USA, lower respiratory tract infection is estimated to cause around 34 deaths per 100,000 children per year and cost 173,000 disability-adjusted life-years per year in children aged <5 years.^4^ However, correctly identifying an ambulant child with pneumonia warranting prescription of antibiotics is difficult.^5–9^ Consequently, overprescription of antibiotics is common.^10–12^ Antibiotics may cause side effects,^13^ increase re-consultation rates,^14^ and repeated use of antibiotics increases antimicrobial resistance in communities and individuals.^15,16^

Diagnostic uncertainty plays an important role in overprescription of antibiotics in children.^10–12,17,18^ Physicians in ambulatory settings still mainly rely on history taking and clinical examination for diagnosis and management decisions. With developing technology, access to additional diagnostic tests in ambulatory settings is increasing, with tests also becoming available as point-of-care tests. For example, point-of-care C-reactive protein (CRP) measurement is increasingly available and recommended by guidelines to aid in diagnosis and management of pneumonia in adults.^19–22^ Although there is currently no accurate biomarker available to distinguish between a bacterial or viral infection,^23^ biomarkers may support correct identification of children at risk of serious infections in ambulatory care.^24^ This might help primary care physicians decide which children with suspected pneumonia need to be more closely monitored or who might benefit from antibiotic treatment.

Systematic reviews have reported separately on the diagnostic value of clinical features^5,25^ and laboratory tests^26^ to identify children with serious infections including pneumonia. These reviews identified shortness of breath, increased work of breathing, and hypoxemia as most important to identify the presence of pneumonia. However, a lack of evidence particularly to identify children with serious infections in primary care was identified. Currently available decision rules for pneumonia showed value in ruling out the need for hospitalization in a child with suspected pneumonia. The rate of false positives when applying this decision rule is high.^27,28^ These decision rules do not aid the physician in identifying the ambulatory child in need of antibiotics. To our knowledge, a systematic review integrating information on all signs, symptoms, and additional tests currently available in ambulatory settings to diagnose pneumonia has not been performed.

The aim of this study was to therefore systematically identify and summarize available evidence of the diagnostic value of signs, symptoms, and additional diagnostic tests for confirming pneumonia or safely ruling it out in ambulant children with signs of a respiratory tract infection in developed countries.

## Results

### Study selection and characteristics

**Fig. 1:**
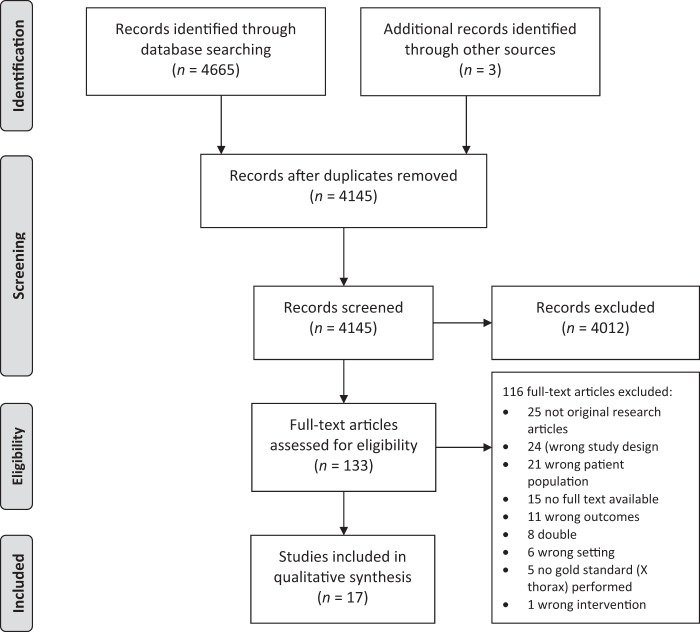
Flow diagram of study selection process

The initial search identified 4306 articles (November 2016) with a further 359 at the November 2017 update. Three studies were identified through reviewing reference lists (Fig. 1). After reviewing titles and abstracts, 133 articles were selected for full text evaluation. Seventeen studies met all the inclusion criteria and were selected for the final data extraction. All selected studies were performed in a secondary care ambulatory setting. Twelve studies reported on signs and symptoms, four on laboratory tests, and six on the use of ultrasonography. Tables 1–3 show the characteristics of the selected studies, with the study of Oostenbrink et al. reporting diagnostic values of index tests in three different patient populations. The prevalence of pneumonia in the selected studies varied from 3.4% to 71.7%. Table 4 shows the outcomes of the quality assessment of the included studies. Risk of bias was present in the majority of the studies in the domain of patient selection. These studies included only children with an indication for chest X-ray (CXR).

**Table 1:** Characteristics of selected articles reporting on signs and/or symptoms

Nr.	Author	Setting	% with pneumonia	Age range	Inclusion criteria	Exclusion criteria	Index test	Reference standard
1	Ayalon et al.^8^	ED, Israel	34%	1 month–16 years	CXR for suspicion of pneumonia	Hospital-acquired pneumonia, aspiration pneumonia	Signs and symptoms	CXR
2	Craig et al.^37^	ED, Australia	3.4%	≤5 years	Fever measured or reported by parents within previous 24 h	Transferred from another hospital, cancer, transplant recipients	Signs and symptoms	CXR
3	Lynch et al.^6^	ED, Canada	35.7%	1–16 years	CXR for suspicion of pneumonia	Chronic respiratory disease, chronic congenital or complex cardiac disease, gastroesophageal reflux, sickle cell disease, malignancy, spastic quadriplegia, asthma requiring >1 bronchodilator treatment at ED, pneumonia in past 8 weeks, critically ill patients	Signs and symptoms, vital signs	CXR
4	Mahabee-Gittens et al.^38^	ED, USA	8.6%	2–59 months	Cough and one of the following: labored, rapid, or noisy breathing; chest or abdominal pain; fever	Currently taking antibiotics; treatment for smoke inhalation, foreign body aspiration, or chest trauma; known diagnoses of asthma, bronchiolitis, cystic fibrosis, sickle cell disease, or chronic cardiopulmonary disease	Signs, symptoms and vital signs	CXR
5	Nijman et al.^39^	ED, the Netherlands	6.7%	1 month–15 years	Fever noted in 24 h before presentation or ≥38.0 °C at ED	Chronic comorbidity, received antibiotics in the past week	Signs and symptoms	CXR, 1-week follow-up
6.1	Oostenbrink et al.^35^	ED, the Netherlands	16%	1 month–16 years	Rectal temperature ≥38.0 °C and cough	Immunodeficiency, multiple handicaps, pre-existing pulmonary disease	Signs, symptoms, vital signs, WBC, CRP	Composite: CXR, or follow-up, or review of records
6.2	Oostenbrink et al.^35^	ED, UK	14%	1 month–16 years	Axillary temperature ≥38.0 °C and lower respiratory tract infection signs	Immunodeficiency	Signs, symptoms, vital signs, WBC, CRP	Composite: CXR, or follow-up, or review of records
6.3	Oostenbrink et al.^35^	ED, UK	3.7%	1 month–16 years	Fever reported or measured rectally ≥38.5 °C and acute breathing difficulty	Immunodeficiency, multiple handicaps, admitted for resuscitation	Signs, symptoms, vital signs, WBC, CRP	Composite: CXR, or follow-up, or review of records
7	Rothrock et al.^29^	ED, USA	20%	≤5 years	Presenting to ED, requiring CXR	CXR obtained for trauma, foreign body ingestion, submersion injury	Canadian task force guideline	CXR
8	Shah et al.^30^	ED; USA	14.5%	≤5 years	CXR for suspicion of pneumonia	CXR for other indications than pneumonia, known illness that placed patient at greater risk of pneumonia	Tachypnea	CXR
9	Shah et al.^34^	ED, US	18%	0–21 years	Clinically suspect of pneumonia, requiring CXR	Hemodynamic instability	Signs and symptoms	CXR
10	Urbankowska et al.^31^	Dept. ped. pulmonology & allergy, Poland	72%	>1 month	Fever >38.0 °C, dyspnea, cough, and abnormal long auscultation	Hospital acquired pneumonia, persistent abnormalities on former CXR, treated for pneumonia in the past 4 weeks	Signs, symptoms, CRP, WBC	CXR
11	Wingerter et al.^32^	ED; USA	16%	≤5 years	CXR for suspicion of pneumonia	Known illness that placed patient at greater risk of pneumonia	WHO criteria for diagnosis of pneumonia	CXR
12	Zukin et al.^33^	ED, USA	14%	<17 years	Clinically assessed need for CXR	None	Signs and symptoms	CXR

*ED* emergency department, *CXR* chest X-ray, *WBC* white blood cell count, *CRP* C-reactive protein

**Table 2:** Characteristics of selected articles reporting on laboratory tests

Nr.	Author	Setting	% with pneumonia	Age range	Inclusion criteria	Exclusion criteria	Index test	Reference standard
5	Nijman et al.^39^	ED, the Netherlands	6.7%	1 month–15 years	Fever noted in 24 h before presentation or ≥38.0 °C at ED	Chronic comorbidity, received antibiotics in the past week	Signs and symptoms, CRP	CXR, 1-week follow-up
6.1	Oostenbrink et al.^35^	ED, the Netherlands	16%	1 month–16 years	Rectal temperature ≥38.0 °C and cough	Immunodeficiency, multiple handicaps, pre-existing pulmonary disease	Signs, symptoms, vital signs, WBC, CRP	Composite: CXR, or follow-up, or review of records
6.2	Oostenbrink et al.^35^	ED, UK	14%	1 month–16 years	Axillary temperature ≥38.0 °C and lower respiratory tract infection signs	Immunodeficiency	Signs, symptoms, vital signs, WBC, CRP	Composite: CXR, or follow-up, or review of records
6.3	Oostenbrink et al.^35^	ED, UK	3.7%	1 month–16 years	Fever reported or measured rectally ≥38.5 °C and acute breathing difficulty	Immunodeficiency, multiple handicaps, admitted for resuscitation	Signs, symptoms, vital signs, WBC, CRP	Composite: CXR, or follow-up, or review of records
10	Urbankowska et al.^31^	Department of pediatric pulmonology and allergy, Poland	72%	>1 month	Fever >38.0 °C, dyspnea, cough, and abnormal long auscultation	Hospital-acquired pneumonia, persistent abnormalities on former CXR, treated for pneumonia in the past 4 weeks	CRP, WBC	CXR
13	Irwin et al.^40^	ED, UK	9.8%	<16 years	Fever, requiring blood tests	Immunodeficiency	CRP, WBC, PCT, resistin, NGAL	CXR

*ED* emergency department, *CXR* chest X-ray, *WBC* white blood cell count, *CRP* C-reactive protein, *PCT* procalcitonin, NGAL neutrophil gelatinase-associated lipocalin

**Table 3:** Characteristics of selected articles reporting on ultrasonography

Nr.	Author	Setting	% with pneumonia	Age range	Inclusion criteria	Exclusion criteria	Index test	Reference standard
9	Shah et al.^34^	ED, US	18%	0–21 years	Clinically suspect of pneumonia, requiring CXR	Hemodynamic instability	Ultrasound	CXR
10	Urbankowska et al.^31^	Dept. ped. pulmonology & allergy, Poland	72%	>1 month	Fever >38.0 °C, dyspnea, cough, and abnormal long auscultation	Hospital-acquired pneumonia, persistent abnormalities on former CXR, treated for pneumonia in the past 4 weeks	Ultrasound	CXR
14	Copetti and Cattarossi^42^	ED, Italy	67%	6 months–16 years	Clinical signs suggesting pneumonia		Ultrasound	CXR
15	Ianniello et al.^43^	ED, Italy	56%	3–16 years	Suspect of pneumonia with fever >38.0 °C for >3 days and cough		Ultrasound	CXR
16	Samson et al.^44^	ED, Spain	42.5%	0–15 years	Suspicion of CAP, performance of CXR	Known pneumonia on arrival to ED, clinical bronchiolitis, previous antibiotic treatment for this episode, chronic chest diseases	Ultrasound	CXR
17	Zhan et al.^41^	Pediatric department, Denmark	50%	0–15 years	Suspicion of pneumonia with cough, fever ≥38.5 °C, elevated respiratory rate, grunting, nasal flaring, or chest recessions or pulse oximetry <95%	Ultrasound not completed within 24 h after inclusion	Ultrasound	CXR

*ED* emergency department, *CXR* chest X-ray, *CAP* community-acquired pneumonia

**Table 4:** Quality assessment according to QUADAS-2

**Nr**.	**Author**	**Patient selection**	**Index test**	**Reference standard**	**Flow and timing**
1	Ayalon (2013)	☹	☺	☺	☺
2	Craig (2010)	☹	☺	☹	☺
3	Lynch (2004)	☹	☺	☺	☺
4	Mahabee (2005)	☺	☺	☺	☺
5	Nijman (2013)	☺	☺	☹	☺
6	Oostenbrink (2013)	☺	☺	☹	☺
7	Rothrock (2001)	☹	☺	☺	☺
8	Shah (2010)	☹	☺	☺	☺
9	Shah (2013)	☹	☺	☺	☺
10	Urbanowska (2015)	☺	☺	☺	☺
11	Wingerter (2012)	☹	☺	☺	☺
12	Zukin (1986)	☹	☺	☺	☺
13	Irwin (2017)	☹	☺	☺	?
14	Copetti (2008)	☹	☺	☹	?
15	Ianniello (2016)	☹	☺	☺	?
16	Samson (2018)	☹	☺	☺	☺
17	Zhan (2016)	☹	☺	☺	☺

☺ low risk of bias ☹ high risk of bias ? unclear risk of bias

### Diagnostic value of signs and symptoms

In total, the included studies reported on 27 signs and symptoms. Separate diagnostic values of all different signs and symptoms are presented in Appendices 2 and 3 for studies with high and low prevalence of pneumonia, respectively. Signs and symptoms that were evaluated in two or more studies are discussed below.

### High prevalence studies

Ten studies reported on the diagnostic value of signs and symptoms in a setting with a pneumonia prevalence >10%.^6,8,29–35^ Nine of these studies reported on tachypnea, but different cutoff points were used. Five studies used the World Health Organization definition of tachypnea.^36^ Four of these studies showed specificity ranging from 73% to 76%, with negative predictive value (NPV) ranging from 85% to 91% (Table 5). Cough was the symptom with the highest reported sensitivity (range 71–88%) (Table 6). Diagnostic value of abnormal auscultation was investigated in several studies. Decreased breath sounds was evaluated in four studies; chest retractions, wheezing, and the presence of crackles in three studies; and the presence of bronchial breathing, rales, and rhonchi in two studies (Table 6). Wheezing, chest retractions, and bronchial breathing showed the highest specificity within this group of symptoms (range 71–96%). Classic signs associated with pneumonia such as crackles or decreased breath sounds showed lower diagnostic performance. An oxygen saturation <94% was assessed in two different studies, with reported specificity ranging between 92% and 96%. Of the more general symptoms, ill appearance was analyzed in two studies and showed the highest NPVs (range 86–92, Table 7).

**Table 5:** Diagnostic value of tachypnea in a setting with high prevalence of pneumonia

Nr.	Author	Pneumonia prevalence	Sensitivity	Specificity	PPV	NPV
6.1	Oostenbrink et al.^35^^a^	14%	59.0	72.8	28.4	90.6
8	Shah et al.^30a^	14.5%	39.7	73.9	20.4	87.9
9	Shah et al.^34^^a^	18%	41	76	27.3	85.4
7	Rothrock et al.^29^^a^	20%	10	5	3	19
11	Wingerter et al.^32a^	16.1%	28.9	75.9	15.9	87.5
1	Ayalon et al.^8^^b^	34%	22.7	89.8	54.0	68.8
3	Lynch et al.^6b^	35.7%	13	95	60	66
6.2	Oostenbrink et al.^35c^	14%	69.0	67.7	25.5	93.2
12	Zukin et al.^33b^	14.4%	50	68	21	89

^a^Cutoff values for tachypnea: WHO definition

^b^Cutoff values for tachypnea: Other

^c^Cutoff values for tachypnea: APLS criteria

*PPV* positive predictive value, *NPV* negative predictive value

**Table 6:** Diagnostic value of assessing breathing and auscultation in a setting with high prevalence of pneumonia

Index test	Study nr.	Pneumonia prevalence	Sensitivity	Specificity	PPV	NPV
Cough	1	34%	78.5	30.2	37.2	72.7
	3	35.7%	88	16	36.8	70.6
Decreased breath sounds	9	18%	24	83	23.7	83.3
	7	20%	9	6	2	20
	3	35.7%	54	55	40,0	68,3
	10	72%	46,1	76,7	83,4	36,0
Wheezing	12	14.4%	6	71	3	82
	3	35.7%	56,0	93	81,7	79,2
	10	72%	3,9	90	49,7	27,00
Crackles	9	18%	24	75	17,4	81,8
	3	35.7%	43	73	47.0	69.7
	10	72%	36.8	60	70.0	27.3
Chest retractions	6.2	14%	56.9	83.4	35.5	92.4
	6.1	16%	28.2	81.5	21.8	86.1
	3	35.7%	5	98	58.2	65.0
Rhonchi	12	14.4%	78	73	15	86
	10	72%	2.6	80	24.8	24.5
Rales	12	14.4%	57	75	27	90
	7	20%	23	15	7	48
Bronchial breathing	3	35.7%	7	96	49.3	65
	10	72%	17.1	93.3	86.6	30.8
Oxygen saturation <94%	6.2	14%	32.8	92.3	40.4	89.5
	6.1	16%	14.1	96.0	39.3	85.9

*PPV* positive predictive value, *NPV* negative predictive value

**Table 7:** Diagnostic value of general symptoms in a setting with high prevalence of pneumonia

Index test	Study nr.	Pneumonia prevalence	Sensitivity	Specificity	PPV	NPV
Ill appearance	6.2	14%	79.3	38.7	17.2	92.1
	6.1	16%	33.3	77.9	21.7	86.4
Fever	12	14.4%	94	36	20	97
	3	35.7%	47	68	45	70
Tachycardia	1	34%	35.6	77.3	45.1	69.6
	3	35.7%	51	60	68	41

*PPV* positive predictive value, *NPV* negative predictive value

### Low prevalence studies

Four studies reported on the diagnostic value of signs and symptoms in settings with a prevalence of pneumonia <10%.^35,37–39^ Diagnostic values of all signs reported in two or more studies are shown in Table 8. Although NPVs were >90% for all evaluated signs and symptoms, the additional diagnostic value of signs and symptoms was low given the low pre-test probability. The absence of tachypnea, crackles, and ill appearance showed the most value when ruling out pneumonia but only decreased the predicted value by 4.8% at most. In some studies, saturation <94%, retractions, and ill appearance gave an increase in positive predictive value (PPV) of up to 27%, but outcomes of different studies varied. As in the high prevalence settings, the classic symptoms associated with pneumonia such as crackles had low diagnostic value.

**Table 8:** Diagnostic value of signs in a low prevalence population with CXR-confirmed pneumonia

Index test	Study nr.	Pneumonia prevalence	Sensitivity	Specificity	PPV	NPV
Tachypnea	5^a^	6.7%	74.27	42.05	8.44	95.78
	6.3^b^	7%	77.78	60.18	13.46	97.14
Tachycardia	2	3.4%	63.04	57.36	4.91	97.80
	5	6.7%	50.29	57.28	7.81	94.12
Saturation <94%	5	6.7%	13.45	97.43	27.38	93.99
	6.3	7%	40.74	88.20	21.57	94.92
Wheezing	5	6.7%	15.38	93.87	8.06	96.95
	4	8.6%	20.45	83.69	10.59	91.76
Crackles	5	6.7%	35.83	92.64	14.54	97.64
	4	8.6%	20.45	86.48	12.50	92.01
Retractions	5	6.7%	14.62	92.51	12.32	93.77
	6.3	7%	29.63	89.38	18.18	94.10
Ill appearance	4	8.6%	31.82	71.24	9.46	91.71
	5	6.7%	30.99	80.01	10.04	94.16
	6.3	7%	77.78	81.42	25.00	97.87

^a^Cutoff values for tachypnea: WHO definition

^b^Cutoff values for tachypnea: APLS criteria

*PPV* positive predictive value, *NPV* negative predictive value

### Diagnostic value of laboratory tests

Four selected studies reported on the diagnostic value of laboratory tests in diagnosing pneumonia (Table 2). Three of these studies were designed to develop a clinical prediction model to identify children with a serious bacterial illness, including pneumonia.^35,39,40^ The clinical prediction models included additional laboratory tests. All four studies showed significantly higher values of CRP and mean white blood cell count (WBC) in the presence of pneumonia. Other laboratory tests were only evaluated in one of the studies or evaluated using different units of measurements. All results are shown in Table 9. The diagnostic models, based first on signs and symptoms, improved when CRP,^35,39^ procalcitonin, and resistin^40^ were added. Data on specific cutoff points for the various laboratory tests and corresponding univariate values for PPV and NPV were not available from any of the selected studies.

**Table 9:** Diagnostic value of laboratory tests for the diagnosis of pneumonia

Pneumonia prevalence	(5) Nijman et al.^39^	(13) Irwin et al.^40^	(6.1) Oostenbrink et al.^35^	(6.2) Oostenbrink et al.^35^	(6.3) Oostenbrink et al.^35^	(10) Urbankowska et al.^31^
	6.7%	11.4%	16%	14%	7%	72%
	Present vs absent	Present vs absent	Present vs absent	Present vs absent	Present vs absent	Present vs absent
Mean CRP (mg/L)	47.5 vs 12.4					
Median CRP (mg/L)		49.0 vs 14.3^a^	86 vs 22^a^	109 vs 31^a^	61 vs 31^a^	1.95 vs 4.7^a^
Mean WBC (×10^9^/L)			17.4 vs 12.8^a^	18.1 vs 12.7^a^	17.0 vs 19.1	
Median WBC (×10^9^/L)		11.8 vs 10.8^a^				
Median WBC count (cells/μL)						10.1 vs 11.3
Median lymphocyte count (cells/μL)						3.6 vs 1.9^a^
Median neutrophil count (×10^9^/L)		8.0 vs 6.2^a^				
Median neutrophil count (cells/μL)						4.3 vs 7.9^a^
NGAL (ng/L)		92.1 vs 69.7^a^				
PCT (µg/L)		0.49 vs 0.18^a^				
Resistin (ng/L)		67.3 vs 35.7^a^				

*CRP* C-reactive protein, *WBC* white blood cell count, *NGAL* neutrophil gelatinase-associated lipocalin, *PCT* procalcitonin

^a^Statistically significant difference

### Diagnostic value of ultrasonography

Six selected studies reported on the diagnostic value of ultrasonography to diagnose pneumonia (Table 3). All these studies had a high prevalence of CXR-confirmed pneumonia (range 18–71.7%). Both PPV and NPV were high in most studies. However, one study found a much lower NPV (ref. ^41^, Table 10). The authors of this paper attributed this difference with previous studies not only to a different study design with better blinding of the sonologist to the patients’ clinical examination in their study but also to the very basic training that pediatric residents received prior to performing the ultrasound examinations in this study.

**Table 10:** Diagnostic value of ultrasonography for the diagnosis of pneumonia

Nr.	Author	Pneumonia prevalence %	Sensitivity	Specificity	PPV	NPV
9	Shah et al.^34^	18	86.1	89.0	63.3	96.7
16	Samson et al.^44^	42.5	87.1	94.8	92.5	90.8
17	Zhan et al.^41^	50	40.2	91.5	82.5	60.5
15	Ianniello et al.^43^	56	97.9	64.9	77.9	96.0
14	Copetti and Cattarossi^42^	67	100	73.1	88.3	100
10	Urbankowska et al.^31^	71.7	93.4	100	100	85.7

*PPV* positive predictive value, *NPV* negative predictive value

## Discussion

### Summary

This clinical systematic review aimed to summarize available evidence on the diagnostic value of relevant clinical information, such as physical signs, laboratory tests, and ultrasonography in the assessment of children suspected of pneumonia in an ambulatory setting. We summarized the results of 17 eligible studies. Overall, diagnosing pneumonia using individual signs and symptoms is not possible. Even established predicting signs of pneumonia, like crackles and tachypnea, score lower than usually presumed.

Although evidence for the diagnostic value of signs and symptoms in the low prevalence settings (prevalence of pneumonia <10%) is limited, clinically ill appearing children and the presence of saturation <94% can support the primary care physician in ruling in pneumonia. In settings with a higher prevalence of pneumonia, the presence of chest retractions, a saturation <94%, and additional tests such as CRP and WBC may further support an accurate diagnosis of pneumonia.

Ultrasonography seems promising given the high PPV and NPV in most of the studies that have evaluated this technique. However, quality assessment did reveal concerns with some of the studies. Specifically, we judged the risk of bias in the domain patient selection high or unclear in many of these studies.^34,41–44^ The high prevalence of pneumonia found in these studies is not representative of most ambulatory settings (Tables 3 and 4). This hampers generalizability, especially to settings with a lower prevalence of pneumonia. Furthermore, ultrasonography requires specific training. Authors describe that training can be basic; however, one study that found a much lower NPV^41^ attributed this partly to the inexperience with ultrasonography of the resident who performed the examination. These findings, combined with the fact that ultrasonography currently may not be available to physicians in primary or even secondary care, makes that adoption of this new technique to ambulatory settings is not evident at this time.

### Strengths and limitations

The field of primary care is evolving quickly, and aside from history taking and clinical examination, primary care physicians increasingly have access to additional diagnostic tools in, or close to, their practice. A strength of our review is that we took this development into account. We conducted a broad search and included all research in developed countries that was aimed at assessing the diagnostic value of history, physical examination, and additional diagnostic tools at the point of care in an ambulatory setting. We specifically chose to only include diagnostic studies performed in developed countries, as we proposed children in developing countries may present with a different range of diseases and more advanced stage of disease at presentation. All search results were evaluated by two authors to ensure that all eligible papers were included. Quality assessment was done systematically by two reviewers using the Quality Assessment of Diagnostic Accuracy Studies 2 (QUADAS-2). Although many studies showed some limitations, most often these limitations seemed inevitable given the chosen study designs or setting.

Our study also has several limitations. We chose to include only studies that used CXR as the golden standard for the diagnosis of pneumonia. Despite known limitations of CXR,^23^ it is still regarded as the reference standard for diagnosis in most studies and also in clinical practice.^23,25^ However, this inclusion criterium may have led to the fact that we found no studies performed in a primary care setting that fulfilled our inclusion criteria. The studies that we did identify were all conducted in ambulatory settings but still prevalence of pneumonia varied greatly. This may be due to differences in organization of health care in different countries. Where some countries offer direct access for patients to secondary care, other countries employ a gate-keeping system with primary care physicians controlling access to secondary care. Which system is used in a country influences the prevalence of pneumonia in children in the secondary care settings. In turn, the prevalence of a disease, and thereby the pre-test probability of being ill or not, is highly relevant for the diagnostic value of a test. A different patient case-mix may lead to so called spectrum bias.^45^

Most studies only included children who had an indication for a CXR. We scored these studies as high risk of bias in the domain patient selection during quality assessment. CXR is recommended only in children who might be admitted to hospital, and are more severely ill, by many guidelines.^3,22,46,47^ Ideally, when assessing the diagnostic value of a test for evaluating children in an ambulatory setting, a study should include all children presenting to a facility without this selection criterion. Only including children with an indication for CXR may mean that these children were at higher risk of actually having pneumonia. This may again bias the diagnostic performance measures.^45^

Furthermore, this review evaluates diagnostic studies. A known limitation of many diagnostic studies is reproducibility, with known low inter-observer agreement between physicians when, for example, assessing auscultation. Whether reporting of signs and symptoms for a study was highly protocolized or not may also have influenced outcomes of individual studies.

We did not perform a meta-analysis in this review as included studies were too heterogeneous in setting, prevalence of pneumonia, and study design to allow pooling of the found results. Rather, we chose to present all the extracted data on diagnostic values. Furthermore, we found no studies that fulfilled our inclusion criteria on the diagnostic value of laboratory tests alone for the diagnosis of pneumonia. So, although selected studies showed that CRP and WBC have diagnostic value, we are unable to calculate this exact value at different cutoff points. Prediction rules combining signs, symptoms, and/or laboratory tests were not included in this review.

### Comparison with other literature

Several systematic reviews have summarized the available evidence on the diagnosis of pneumonia in children. These reviews have all mainly focused on the objective to correctly identify the child who suffers from pneumonia. Rambaud-Althaus et al.^48^ and Shah et al.^25^ evaluated the diagnostic value of clinical features in younger children, also including children in low resource settings. Rambaud-Althaus et al.^48^ specifically examined studies in children aged <5 years. Like in our review, both found large heterogeneity and were unable to define one clinical feature for the diagnosis of pneumonia. Highest positive likelihood ratios were found for respiratory rate over 50 breaths per minute,^48^ hypoxemia, and increased work of breathing.^25^ Shah et al. also found that tachypnea has diagnostic value in excluding pneumonia. Both reviews stress the importance of combining the different features to make the diagnosis of pneumonia more or less likely and recommend further investigation of these combinations and possibly adding point-of-care tests. A systematic review by van den Bruel et al.^5^ focused on clinical features to identify serious bacterial illness, including pneumonia, in children in developed countries. They found that respiratory rate was the most reliable sign in the diagnosis of pneumonia (positive likelihood ratio of 2.7–4.0 dependent on the cutoff point), but breathlessness and auscultatory signs did not have high diagnostic values. Another systematic review by van den Bruel et al. looked specifically at the diagnostic value of laboratory tests in identifying children with serious infections, including pneumonia.^26^ They found that measuring CRP and procalcitonin provided most diagnostic value, and logically different cutoff points were necessary to either rule-in or rule-out serious infection. Diagnostic value specifically for pneumonia was not available from this review. As in our review, the lack of studies in settings with low prevalence of serious infections is identified as a gap in the current evidence, as well as in studies combining signs, symptoms, and laboratory tests.

A recent review looking at the advances in the diagnosis of pneumonia by Zar et al.^23^ summarizes some of the newer tests. Many of the described possible new biomarkers in this article, which mainly aim to differentiate between bacterial and viral infection, are at this stage far from being available as point-of-care tests in an ambulatory setting and therefore fall out of the scope of our article. However, they also describe the growing body of evidence on ultrasonography for the diagnosis of pneumonia, a technique that is already available using a point-of-care device. They conclude that, although this new technique seems promising, the effect on clinical outcomes in patients warrants further investigation. We feel that this is a very relevant recommendation, also supported by the results found in our review. Further research is also necessary in settings with a lower prevalence of pneumonia to evaluate whether this technique holds possibility for adoption to such settings.

### Implications for practice and further research

Not a single clinical sign or symptom has sufficient diagnostic value to adequately diagnose pneumonia in children or safely rule it out. Dependent on the setting, the physician needs to combine multiple features to make the diagnosis more or less likely. When assessing the results and applying them in practice, it is important that physicians are aware of the prevalence of pneumonia in their own setting and keep in mind that diagnostic value of tests may be different in their setting. The prevalence of pneumonia in the included studies in this review is stated in different tables in our review, and studies with a lower prevalence of pneumonia (<10%) are presented separately from the studies with higher prevalence of pneumonia. Primary care physicians may evaluate the results presented for low prevalence settings but should be conscious of the fact that this evidence was derived from trials in secondary ambulatory care settings.

Future research should focus on the combination of clinical features and additional testing as the latter becomes more widely available. This can be done by designing new diagnostic trials but combining the already available evidence using the technique of meta-analysis of individual data may also increase our knowledge. In adults, this method has, for example, increased our knowledge of the added value of newer additional tests in ambulatory care like CRP.^49^

There is a lack of evidence concerning the diagnosis of pneumonia in primary care settings with low prevalence of pneumonia. More research should be conducted in this setting, as many children are evaluated in primary care, and a large proportion of overprescription of antibiotics occurs in this setting.^10–12^ The role of additional tests such as point-of-care biomarkers or ultrasound should be subject of further evaluation leading to evidence that can help the primary care physician determine which children are in need of antibiotic treatment. In these settings, it is especially important for a physician to rule out pneumonia safely. This means the NPV of an index test is of major importance in this setting.

In conclusion, not a single item from history taking, physical examination, or additional laboratory testing is sufficient to diagnose pneumonia in ambulant children and combining tests will be necessary to increase diagnostic certainty. Very few diagnostic studies are conducted in settings with low prevalence of pneumonia. Future research should focus on increasing evidence for these settings and on synthesizing the evidence currently available. The addition of diagnostic tests seems promising for the future, but further evaluation is needed. Furthermore, clinical implications of additional diagnostic testing, including possibly performing ultrasound evaluation, also need careful evaluation.

## Methods

### Search strategy

We performed a systematic search for articles reporting the diagnostic value of items from history and/or physical examination and/or additional diagnostic tests for diagnosing pneumonia. We searched the databases PubMed and Embase without restriction in date of publication and language. Search terms included Mesh terms and free text terms on respiratory tract infection, pneumonia, clinical and laboratory test, infant or child, and ambulatory care or primary care. Full search strategy is listed in Appendix 1. The first search was undertaken in November 2016 and was updated in November 2017. We checked reference lists of all retrieved articles. The study was registered with PROSPERO (https://www.crd.york.ac.uk/PROSPERO/display_record.php?RecordID=60439).

### Study selection

Two independent reviewers performed selection. Selection was done in two rounds, first based on titles and abstracts (performed by W.G.G. and M.J.C.S.), and in the second round, based on the full text article (performed by A.R.J.D. and M.J.C.S.). In case of doubt or conflict, a third reviewer (J.W.L.C.) was consulted.

Inclusion criteria for further evaluation are summarized in Box 1. We only included studies on children aged 0–12 years. Pneumonia was defined as radiological-confirmed pneumonia on CXR. We only included index tests that are currently available as point-of-care tests or have the possibility to soon be available as such a test, following the international consented definition for point-of-care tests in a primary care: “a test to support clinical decision making, which is performed by a qualified member of the practice staff nearby the patient and on any part of the patient’s body or its derivatives, during or very close to the time of consultation, to help the patient and physician to decide upon the best suited approach, and of which the results should be known at the time of the clinical decision making”.^50^ Further specified inclusion criteria were formulated for study design, population and setting; reported outcomes; and use of index tests and reference standard (Box 1).

### Box 1 Criteria for study selection

**Design**

Studies that prospectively assessed diagnostic accuracy were selected. Narrative reviews, letters, editorials, comments, and case series of less than 20 patients were excluded. Systematic reviews and meta-analyses were used only as a source of references.

**Population**

Studies needed to include children (0-12) with suspected pneumonia. If the study consisted only partially of children, results for children needed to be reported separately. Studies concerning only neonates were excluded.

**Setting**

Studies were performed in ambulatory setting in developed countries i.e. general practice, out-of-hours clinic, emergency room or the outpatient department of the hospital. Studies done in developing countries were excluded because of the different range of diseases and more advanced stage of disease at presentation. We used the United Nations list to define developed countries, which include Europe, Canada, the USA, Australia, New Zealand, and Japan.

**Target disease and reference standard**

Selected studies assessed the detection of community acquired pneumonia. Pneumonia was confirmed by chest X-ray.

**Index test**

Studies that assessed signs, symptoms, additional test and/or biomarkers to predict the presence of pneumonia were selected. Studies reporting on additional test and/or biomarkers were selected only if they were available in an ambulant setting or could become readily available in the near future.

**Data reporting**

Diagnostic value of signs, symptoms or additional tests was reported in the article or could be calculated from the data in the article

### Quality assessment

The methodological quality of eligible full text articles was evaluated independently by two reviewers (A.R.J.D. and M.J.C.S.) using the QUADAS-2 tool.^51^ In case of doubt or disagreement, a third reviewer was consulted (J.W.L.C.). In the assessment of risk of bias related to patient selection, we presumed the risk of bias to be high when only children who had a clinical indication for a CXR were selected for inclusion.

### Data extraction

One author (M.J.C.S.) extracted the data. Study characteristics (year of publication, study setting, age of study population, percentage of children with pneumonia, and total size of study population) and use of reference test and index tests (definitions, procedures) were noted on predefined forms. All clinical evaluations and all laboratory tests or imaging techniques that are available for point-of-care testing or may be available in ambulant care in the near future were considered as index tests and data on each index test were individually extracted per article. Signs and symptoms with different cutoff values were considered as separate index tests. Combinations of tests and clinical prediction rules were not considered in this review.

### Analysis

From the raw data extracted from each study, we constructed two-by-two tables and calculated the relevant measures of diagnostic accuracy, i.e. sensitivity, specificity, PPV, and NPV for each individual index test. When extracted data were insufficient to make these calculations, authors from the original studies were contacted and asked to provide more details. Given the aim of our study, results include both NPV and PPV. In a setting of low prevalence of pneumonia such as primary care, ruling out pneumonia is most relevant, making the NPV a more important diagnostic value of a test. In a setting such as an emergency care, both values may be of equal importance, but priority may be on ruling in pneumonia. This makes the PPV of the index test of higher importance.

Because of the wide range of pneumonia prevalence in different settings,^25,48^ diagnostic values of individual index tests are presented separately for studies with low prevalence of pneumonia (≤10%) and for studies with a high prevalence of pneumonia (>10%). With expected high heterogeneity between studies, we planned meta-analysis if at least four studies with comparable inclusion criteria and comparable settings reported on an index test. After reviewing all included studies, meta-analysis was not possible.

## Electronic supplementary material

Appendix 1

## Data Availability

The manuscript is based fully on previously published articles.^6,8,29–35,37–44^ No data sets were generated or analyzed during the current study. Additional information on calculations performed with extracted data is available upon request to the corresponding author.

## Electronic supplementary material

**Supplementary information** accompanies the paper on the *npj Primary Care Respiratory Medicine* website (10.1038/s41533-018-0104-8).
