# Supplementary material for: Diagnostic value of signs, symptoms and diagnostic tests for diagnosing pneumonia in ambulant children in developed countries: a systematic review
Source: NPJ Prim Care Respir Med. 2018 Oct 26;28:40. doi: 10.1038/s41533-018-0104-8 (PMC6203790; doi:10.1038/s41533-018-0104-8)
Supplement: Supplementary file 1 — Appendix 1 [file 41533_2018_104_MOESM1_ESM.pdf]

Appendix 1 Full search criteria for PubMed and Embase

Search terms PubMed:
Concerning diagnosis:
"Respiratory Tract Infections"[Mesh] OR “pneumonia”[MeSH terms] OR Pneumonia/ OR Pneumonia, Bacterial/ OR (community-acquired pneumon*[tiab] or community acquired pneumon*[tiab])

Concerning diagnostic features:
"Signs and Symptoms"[MeSH] OR signs and symptoms OR "Fever"[MeSH] OR fever OR fast breathing OR tachypnea OR respiratory rate OR yale observation scale OR yale score OR yale scale OR Nelson score OR Nelson scale OR young infant observation scale OR "Tachycardia"[Mesh] OR fast heart rate OR “capillary refill time” OR clinical assessment*[tiab] OR clinical feature*[tiab] OR “predictive value of tests”[MeSH terms] OR “sensitivity and specificity”[MeSH terms] OR “reproducibility of results”[MeSH terms] OR “diagnostic test” OR “diagnostic tests” OR “physical examination”[MeSH terms] OR “medical history taking”[MeSH terms] OR "Clinical Laboratory Techniques"[Mesh]

Concerning the study population:
"infant"[MeSH Terms] OR "child"[MeSH Terms] OR "adolescent"[MeSH Terms] OR paediatric [All fields] OR pediatric [All fields] OR “pediatrics” [MeSH term]

Concerning the study setting:
"Ambulatory Care"[Mesh] OR "Family Practice"[Mesh] OR general practice OR GP OR "Physicians, Family"[Mesh] OR "Primary Health Care"[Mesh] OR "Emergency Service, Hospital"[Mesh] OR primary care

Search terms Embase:
Concerning diagnosis:
‘pneumonia’ OR ‘lower respiratory tract infection’ OR ‘respiratory tract infection’ OR 'respiratory tract infection'/exp OR ‘infectious pneumonia’/exp OR 'lung infiltrate'/exp OR 'community acquired pneumonia'/exp

Concerning diagnostic features:
‘diagnostic accuracy’/exp OR ‘predictor variable’/exp OR ‘breathing rate’/exp OR 'physical disease by body function'/exp OR (signs AND symptoms) OR 'fever'/exp OR fever OR ‘fast breathing’ OR tachypnoea OR ‘respiratory rate’ OR 'tachycardia'/exp OR ‘fast heart rate’ OR ‘capillary refill’ OR 'blood analysis’ OR ‘laboratory analysis' OR 'anamnesis'/exp OR 'blood examination'/exp OR 'examination'/exp OR 'respiratory tract examination'/exp OR 'vital sign'/exp OR 'lung auscultation'/exp OR 'diagnostic test'/exp OR 'diagnostic value'/exp OR 'diagnostic test accuracy study'/exp OR 'diagnostic value'/exp OR 'echography'/exp OR 'laboratory diagnosis'/exp OR 'physical examination'/exp OR 'symptom assessment'/exp

Concerning the study population:
'infant'/exp OR 'preschool child'/exp OR 'school child'/exp OR 'toddler'/exp OR 'adolescent'/exp OR 'pediatrics'/exp

Concerning the study setting:
'ambulatory care'/exp OR 'general practice'/exp OR ‘general practice’ OR gp OR 'general practitioner'/exp OR ‘family physician’ OR 'primary medical care'/exp OR ‘primary care’ OR 'emergency ward'/exp

Appendix 2 Diagnostic value of signs & symptoms in a high prevalence population with CXR confirmed pneumonia

		Ayalon (2013)				Lynch (2004)				Oostenbrink (2013)				Oostenbrink (2013)				Rothrock (2001)				Shah (2010)				Shah (2013)				Urbankowska (2015				Wingerter 2012				Zukin (1986)			
Pneumonia prevalence		181/525 (34%)				204/571 (35,70%)				Pop 1: 78/504 (16%)				Pop 2: 58/420 (14%)				67/329 (20%)				235/1622 (14.5%)				36/200 (18%)				76/106 (72%)				324/2008 (16.1%)				18/125 (14.4%)			
		Sensitivity	Specificity	PPV	NPV	Sensitivity	Specificity	PPV	NPV	Sensitivity	Specificity	PPV	NPV	Sensitivity	Specificity	PPV	NPV	Sensitivity	Specificity	PPV	NPV	Sensitivity	Specificity	PPV	NPV	Sensitivity	Specificity	PPV	NPV	Sensitivity	Specificity	PPV	NPV	Sensitivity	Specificity	PPV	NPV	Sensitivity	Specificity	PPV	NPV
Symptom																																									
Rhinorrhea		49.72	46.22	32.73	63.60	5.00	96.00	41.00	64.51																																
Cough		78.45	30.23	37.17	72.73	88.00	16.00	36.80	70.58																													50	77	27	90
Emesis		29.83	74.42	38,03	66.84																																				
Diarrhea		9.39	90.41	34.00	65.47																																				
Chills		9.39	95.93	54.84	66.80																																				
Weakness		8.33	96.22	53.57	66.73																																				

		Ayalon (2013)				Lynch (2004)				Oostenbrink (2013)				Oostenbrink (2013)				Rothrock (2001)			
Pneumonia prevalence		181/525 (34%)				204/571 (35,70%)				Pop 1: 78/504 (16%)				Pop 2: 58/420 (14%)				67/329 (20%)			
		Sensitivity	Specificity	PPV	NPV	Sensitivity	Specificity	PPV	NPV	Sensitivity	Specificity	PPV	NPV	Sensitivity	Specificity	PPV	NPV	Sensitivity	Specificity	PPV	NPV
Dyspnea		16.57	90.70	48.39	67.39	5.00	96.00	41.00	64.51												
History of fever						92.00	21.00	39.30	82.52												
Pleuritic pain						3.00	94.00	21.75	63.55												
Wheeze						4.00	97.00	42.57	64.51												
Sign																					
Tachycardia		35.56	77.33	45.07	69.63	51	60	68	41												
Tachypnea		22.65	89.83	53.95	68.82	13	95	60	66	58.97	72.77	28.40	90.64	68.97	67.68	25.48	93.16	10	5	3	19

		Shah (2010)				Shah (2013)				Urbankowska (2015				Wingerter 2012				Zukin (1986)			
Pneumonia prevalence		235/1622 (14.5%)				36/200 (18%)				76/106 (72%)				324/2008 (16.1%)				18/125 (14.4%)			
		Sensitivity	Specificity	PPV	NPV	Sensitivity	Specificity	PPV	NPV	Sensitivity	Specificity	PPV	NPV	Sensitivity	Specificity	PPV	NPV	Sensitivity	Specificity	PPV	NPV
Dyspnea																					
History of fever																					
Pleuritic pain																					
Wheeze																					
Sign																					
Tachycardia																					
Tachypnea		39.66 (WHO)	73.93	20.40	87.91	41	76	27.27	85.44					28.9	75.9	15.9	87.5	50	68	21	89

		Ayalon (2013)				Lynch (2004)				Oostenbrink (2013)				Oostenbrink (2013)				Rothrock (2001)			
Pneumonia prevalence		181/525 (34%)				204/571 (35,70%)				Pop 1: 78/504 (16%)				Pop 2: 58/420 (14%)				67/329 (20%)			
		Sensitivity	Specificity	PPV	NPV	Sensitivity	Specificity	PPV	NPV	Sensitivity	Specificity	PPV	NPV	Sensitivity	Specificity	PPV	NPV	Sensitivity	Specificity	PPV	NPV
Oxygen saturation	>95%	84.09	4.44	31.42	34.88																
	90-95%	15.34	95.86	65.85	68.50																
	<90%	0.57	99.70	50.00	65.82																
	<94%									14.10	96.01	39.29	85.92	32.76	92.27	40.43	89.54				
Ill appearance										33.33	77.93	21.67	86.46	79.31	38.67	17.16	92.11				
Wheezing						56.02	93.00	81.65	79.18												
Rhonchi																					

		Shah (2010)				Shah (2013)				Urbankowska (2015				Wingerter 2012				Zukin (1986)			
Pneumonia prevalence		235/1622 (14.5%)				36/200 (18%)				76/106 (72%)				324/2008 (16.1%)				18/125 (14.4%)			
		Sensitivity	Specificity	PPV	NPV	Sensitivity	Specificity	PPV	NPV	Sensitivity	Specificity	PPV	NPV	Sensitivity	Specificity	PPV	NPV	Sensitivity	Specificity	PPV	NPV
Oxygen saturation	>95%																				
	90-95%																				
	<90%																				
	<94%																				
Ill appearance																					
Wheezing										3.90	90.00	49.70	26.99					6	71	3	82
Rhonchi										2.60	80.00	24.77	24.48					78	73	15	86

	Ayalon (2013)				Lynch (2004)				Oostenbrink (2013)				Oostenbrink (2013)				Rothrock (2001)				Shah (2010)				Shah (2013)				Urbankowska (2015				Wingerter 2012				Zukin (1986)			
Pneumonia prevalence	181/525 (34%)				204/571 (35,70%)				Pop 1: 78/504 (16%)				Pop 2: 58/420 (14%)				67/329 (20%)				235/1622 (14.5%)				36/200 (18%)				76/106 (72%)				324/2008 (16.1%)				18/125 (14.4%)			
	Sensitivity	Specificity	PPV	NPV	Sensitivity	Specificity	PPV	NPV	Sensitivity	Specificity	PPV	NPV	Sensitivity	Specificity	PPV	NPV	Sensitivity	Specificity	PPV	NPV	Sensitivity	Specificity	PPV	NPV	Sensitivity	Specificity	PPV	NPV	Sensitivity	Specificity	PPV	NPV	Sensitivity	Specificity	PPV	NPV	Sensitivity	Specificity	PPV	NPV
Rales																	23	15	7	48																	57	75	27	90
Crackles					43.00	73.00	46.96	69.73																	24	75			36.80	60.00	69.98	27.26								
Bronchial breathing					7.00	96.00	49.31	65.00																					17.10	93.30	86.61	30.76								
Decreased breath sounds					54.00	55.00	40.01	68.26									9	6	2	20					24	83			46.10	76.70	83.37	35.97								
Retractions					5.00	98.00	58.15	64.98																																
Grunting					2.00	100.00	100.00	64.74																																
Fever					47	68	45	70																													94	36	20	97

		Ayalon (2013)				Lynch (2004)				Oostenbrink (2013)				Oostenbrink (2013)				Rothrock (2001)				Shah (2010)				Shah (2013)				Urbankowska (2015				Wingerter 2012				Zukin (1986)			
Pneumonia prevalence		181/525 (34%)				204/571 (35,70%)				Pop 1: 78/504 (16%)				Pop 2: 58/420 (14%)				67/329 (20%)				235/1622 (14.5%)				36/200 (18%)				76/106 (72%)				324/2008 (16.1%)				18/125 (14.4%)			
		Sensitivity	Specificity	PPV	NPV	Sensitivity	Specificity	PPV	NPV	Sensitivity	Specificity	PPV	NPV	Sensitivity	Specificity	PPV	NPV	Sensitivity	Specificity	PPV	NPV	Sensitivity	Specificity	PPV	NPV	Sensitivity	Specificity	PPV	NPV	Sensitivity	Specificity	PPV	NPV	Sensitivity	Specificity	PPV	NPV	Sensitivity	Specificity	PPV	NPV
Chest retractions/nasal flare										28.21	81.46	21.78	86.10	56.90	83.43	35.48	92.35																								
Respiratory distress																		25	18	7	48																	17	84	15	86

Cut-of values for tachypnoea: [1]WHO definition [2]APLS criteria [3]other

Abbreviations:

PPV: positive predictive value, NPV: negative predictive value, Pop: population

Appendix 3 Diagnostic value of signs & symptoms in a low prevalence population with CXR confirmed pneumonia

		Craig (2010)				Mahabee (2005)				Nijman (2013)				Oostenbrink (2013)			
Pneumonia prevalence		3.4% (533/15781)				8.6% (44/510)				(6.7%) 171/2547				Pop 3 27/366 (7%)			
		Sensitivity	Specificity	PPV	NPV	Sensitivity	Specificity	PPV	NPV	Sensitivity	Specificity	PPV	NPV	Sensitivity	Specificity	PPV	NPV
Symptom																	
Cough		86.87	47.26	5.44	99.04												
Diarrhea		21.76	74.36	2.88	96.46												
Dyspnea/difficult breathing		45.40	87.46	11.22	97.87												
Crying		37.15	67.13	3.80	96.84												
Sign																	
Tachycardia		63.04	57.36	4.91	97.80					50.29	57.28	7.81	94.12				

		Craig (2010)				Mahabee (2005)				Nijman (2013)				Oostenbrink (2013)			
Pneumonia prevalence		3.4% (533/15781)				8.6% (44/510)				(6.7%) 171/2547				Pop 3 27/366 (7%)			
		Sensitivity	Specificity	PPV	NPV	Sensitivity	Specificity	PPV	NPV	Sensitivity	Specificity	PPV	NPV	Sensitivity	Specificity	PPV	NPV
Tachypnoea										74.27	42.05	8.44	95.78	77.78	60.18	13.46	97.14
Oxygen saturation	<94%									13.45	97.43	27.38	93.99	40.74	88.20	21.57	94.92
Ill appearance										30.99	80.01	10.04	94.16	77.78	81.42	25.00	97.87
Ill appearance	Well	18.95	58.38	1.56	95.38												
	Mildly unwell	54.03	50.31	3.66	96.91												
	Moderately unwell	24.20	91.63	9.17	97.19												
	Very unwell	2.81	99.68	23.44	96.71												

		Craig (2010)				Mahabee (2005)				Nijman (2013)				Oostenbrink (2013)			
Pneumonia prevalence		3.4% (533/15781)				8.6% (44/510)				(6.7%) 171/2547				Pop 3 27/366 (7%)			
		Sensitivity	Specificity	PPV	NPV	Sensitivity	Specificity	PPV	NPV	Sensitivity	Specificity	PPV	NPV	Sensitivity	Specificity	PPV	NPV
Wheezing		15.38	93.87	8.06	96.95	20.45	83.69	10.59	91.76								
Crackles		35.83	92.64	14.54	97.64	20.45	86.48	12.50	92.01								
Decreased breath sounds						11.36	94.85	17.24	91.89								
Abnormal chest sounds		54.03	85.70	11.60	98.17												
Rash		9.01	82.12	1.73	96.28												
Capp refill >2sec		12.01	95.72	8.91	96.89												
Capp refill >3sec										4.09	96.42	7.61	93.32				

		Craig (2010)				Mahabee (2005)				Nijman (2013)				Oostenbrink (2013)			
Pneumonia prevalence		3.4% (533/15781)				8.6% (44/510)				(6.7%) 171/2547				Pop 3 27/366 (7%)			
		Sensitivity	Specificity	PPV	NPV	Sensitivity	Specificity	PPV	NPV	Sensitivity	Specificity	PPV	NPV	Sensitivity	Specificity	PPV	NPV
Temperature ≥38°C		82.55	21.95	3.56	97.30												
Temperature ≥39°C		54.97	57.89	4.36	97.36												
Nasal flaring						22.73	92.27	21.74	92.67								
Grunting						2.27	95.92	5.00	91.22								
Retractions						31.82	71.24	9.46	91.71	14.62	92.51	12.32	93.77	29.63	89.38	18.18	94.10

Appendix 4 Diagnostic value of ultrasonography

	Shah (2013)				Samson (2018)				Zhan (2016)				Urbankowksa (2015)				Ianniello (2015)				Copetti (2008)			
% Final diagnosis pneumonia	36/200 (18%)				85/200 (42.5%)				82/164 (50%)				76/106 (71.7%)				47/84 (56%)				53/79 (67%)			
	Sensitivity	Specificity	PPV	NPV	Sensitivity	Specificity	PPV	NPV	Sensitivity	Specificity	PPV	NPV	Sensitivity	Specificity	PPV	NPV	Sensitivity	Specificity	PPV	NPV	Sensitivity	Specificity	PPV	NPV
Point-of-care ultrasonography	86.11	89.02	63.27	96.69	87.1	94.8	92.5	90.8	40.2	91.5	82.5	60.5	93.4	100	100	85.7	97.87	64.86	77.97	96.00	100.00	73.08	88.33	100.00
> 1cm lung consolidation on ultrasonography	86	97																						
Right chest									49	88														
Left chest									33	95														
